# Tuberculous Disease of the Spinal Marrow

**Published:** 1843-04

**Authors:** 


*Tuberculous Disease of the Spinal Marrow.*—Dr. J. B. S. Jackson exhibited a specimen of the above disease. It was situated at the sixth cervical vertebra, and involved almost the entire substance of the organ at that part, forming an opaque, yellowish, solid, well-defined and uniform mass about as large as the tip of the little finger. The spinal marrow was somewhat enlarged at the seat of disease, and was a little softened just above and below it, but was elsewhere quite healthy. The membranes, also, were healthy, except for some thickening of the dura mater at the upper part. In the brain there was a copious effusion of serum, with complete softening of the septum. Extensive tuberculous disease was found in both lungs and in the intestines, besides similar disease in the prostate gland and in the kidneys.

The patient, an Irish laborer, æt. 42, died September 25th. Health quite good previously to the last year, and since then no local trouble except a disease in the ankle joint. The symptoms of spinal disease came on about three and a half months before death, and, when examined on the 13th of August, were as follows: diminished sensation, with hardly a trace of voluntary motion in the lower extremities, the upper being very much less affected; very frequent and involuntary contractions of the right lower extremity, tending to draw the limb up into a strongly flexed position, and attended with very severe pain; some contractions of the left lower extremity, but without pain. Even on moving the bed clothes, the muscles of the lower extremities would be strongly excited, and, in attempting to straighten them when they were drawn up, his suffering was very great. In the upper extremities these spasms were very much less. The bladder was completely paralyzed, so that the catheter had been used daily for the last three weeks. These symptoms continued with but little change till death, the spasms being on one occasion so violent that the patient was fairly jerked out of bed, and fell upon the floor, though fortunately with but little injury. He suffered much also from a morbid sesibility, and from neuralgic pains in the right lower extremity, passing up into the abdomen. Respiration was carried on by the diaphragm, the intercostal muscles seeming to be completely paralyzed. The catheter was used till the last month, after which the urine became involuntary; the dejections, also, were for the most part involuntary, whenever procured, the bowels being very costive. The spine was often examined, but the patient scarcely ever allowed any pain or tenderness there, neither was there any trouble in the head worth mentioning. When first seen his general aspect was sufficiently well, but as the disease advanced, he became exceedingly emaciated, and for sometime before death was very much sunken, with sloughs about the sacrum. As to his pulmonary disease, he had no symptoms that led to a suspicion of it; these was occasionally some dyspnœa, but, as it was generally accompanied with a feeling as of a cord about the lower part of the chest, it was attributed to the paralysis; the nurse, on being questioned after the death of the patient, mentioned an occasional, very slight cough during the last few days, but never before.—*Rep. of Bost. Soc. for Med. Improvement*, Sept. 26th, 1842, in *N. E. Quarterly.*

